# Persistent thrombocytosis in β-thalassemia post-splenectomy: A STROBE-compliant retrospective cohort study at a Jordanian referral center

**DOI:** 10.1097/MD.0000000000048717

**Published:** 2026-05-15

**Authors:** Aisheh Alesufi, Naser Aldain A. Abu Lehyah, Dima Abu Nasrieh, Haneen A. Banihani, Saif Aburumman, Zahraa Zibara, Nour Fakih, Lama Al-Karmi, Maram A. Al Nawaiseh, Qasem Shersheer

**Affiliations:** a Thalassemia and Hemophilia Center, Women’s and Children’s Hospital – Albashir Hospitals, Amman, Jordan; b Division of Neonatology, Department of Pediatrics, Women’s and Children’s Hospital – Albashir Hospitals, Amman, Jordan; c Jordan University Hospital, Amman, Jordan; d Gilbert and Rose-Marie Chagoury School of Medicine, Lebanese American University, Byblos, Lebanon; e School of Dental Medicine, BAU International University Batumi, Batumi, Georgia; f Health Data Analytics Department, Electronic Health Solutions, Amman, Jordan.

**Keywords:** β-thalassemia, blood transfusion, hematological disorder, Jordan, splenectomy, thrombocytosis

## Abstract

Beta-thalassemia is a prevalent autosomal recessive hematological disorder characterized by defective β-globin chain production. Splenectomy is commonly performed in severe cases to alleviate transfusion dependency, but persistent post-splenectomy thrombocytosis poses significant clinical challenges. This Strengthening the Reporting of Observational Studies in Epidemiology-compliant retrospective cohort study included β-thalassemia major patients who underwent splenectomy at Al-Bashir Hospital between 2018 and 2024. Hematologic and biochemical parameters before and 1 year after surgery were compared using paired *t* tests after verifying data normality with the Shapiro–Wilk test (*P* > .05). A *P* value < .05 was considered statistically significant. Persistent thrombocytosis was observed in all patients, with mean platelet counts of 965.36 ± 413.50 × 10^9^/L immediately after surgery, decreasing slightly to 843.23 ± 320.08 × 10^9^/L at the 1-year follow-up. Hemoglobin levels significantly increased from 8.91 ± 0.96 g/dL pre-splenectomy to 9.65 ± 1.49 g/dL post-splenectomy. Blood transfusion requirements decreased by 42.49%, and transfusion intervals lengthened significantly, confirming the procedure’s effectiveness in reducing transfusion burden. Ferritin levels declined, reflecting improved iron overload management. Nevertheless, adherence to postoperative medications, including aspirin and hydroxyurea, was suboptimal, potentially elevating thrombotic risk. Splenectomy in β-thalassemia major is associated with sustained hematologic improvement but persistent thrombocytosis. These findings underscore the importance of long-term platelet monitoring and strict adherence to antithrombotic prophylaxis to ensure optimal safety and clinical outcomes. Further multicenter studies are warranted to establish standardized monitoring and management protocols for this population.

## 1. Introduction

Thalassemia, one of the most common forms of inherited anemia worldwide, is characterized by decreased or impaired synthesis of one of the globin chains required to produce hemoglobin.^[1]^ A defect in producing the β-globin gene results in β-thalassemia, which is clinically classified into 3 subtypes: β-thalassemia major, β-thalassemia intermedia, and β-thalassemia minor.^[2,3]^ In 2021, the worldwide number of thalassemia cases was 1,310,407.^[4]^ More than 200 β-thalassemia-causing genetic mutations have been identified.^[2]^ The frequency of β-thalassemia carriers varies geographically, with particularly high rates in the Middle East, the Mediterranean, and Central and Eastern Asia.^[5]^ For instance, in the Middle East, carrier prevalence can reach 15%, largely due to consanguineous marriages, which average between 25% and 60% in the region.^[5]^

In Jordan, β-thalassemia carriers are estimated to range between 2% and 4%, with approximately 1450 patients registered as of 2019, of whom 1228 were receiving treatment.^[5]^ To reduce the burden of hemoglobinopathies, Jordan implemented mandatory premarital genetic screening in 2007.^[6]^ However, β-thalassemia major, also referred to as transfusion-dependent thalassemia, continues to pose significant challenges, as it relies on regular transfusion therapy to maintain hemoglobin levels between 9.5 and 10.5 g/dL.^[7]^ While transfusion therapy effectively suppresses ineffective erythropoiesis, it is associated with complications such as iron overload, necessitating regular monitoring and chelation therapy.^[7]^

Splenectomy is a key intervention for β-thalassemia patients whose annual blood requirements exceed 1.5 times those of non-splenectomized patients, or who present with symptomatic splenomegaly or hypersplenism causing leukopenia or thrombocytopenia.^[8]^ While splenectomy reduces transfusion requirements, it also carries significant risks, including sepsis and thrombocytosis.^[9]^ Thrombocytosis, defined as a platelet count exceeding 450,000/μL, is observed in up to 75% of splenectomized patients without preexisting myeloproliferative disorders.^[10,11]^

Another complication following splenectomy is reactive thrombocytosis, which occurs in approximately 78% of cases, of whom around 5% develop thrombosis postoperatively.^[9]^ A study conducted on 129 splenectomized patients, regardless of the underlying cause, found that 95% of patients had their platelet counts return to the normal range within 1 to 5 years post-splenectomy.^[12]^ Secondary thrombocytosis, which resolves once the underlying cause is addressed, is far more common than primary thrombocytosis.^[11]^ However, extreme thrombocytosis – platelet counts exceeding 1,000,000/μL – can lead to thromboembolic complications, as evidenced by its occurrence in 19% of post-splenectomy or hyposplenism patients.^[13]^ These risks highlight the need for long-term monitoring and preventive measures, such as vaccinations and antibiotic prophylaxis against encapsulated pathogens, including *Streptococcus pneumoniae*, *Haemophilus influenzae*, and *Neisseria meningitidis*.^[8]^

Despite extensive research on β-thalassemia, studies addressing post-splenectomy outcomes, particularly thrombocytosis, remain scarce. Most available data focus on generalized complications rather than specific regional insights. To the best of our knowledge, this is the first study in Jordan to comprehensively evaluate post-splenectomy complications in β-thalassemia patients. The primary endpoint of our study was platelet count 1 year post-splenectomy, with multiple secondary endpoints, including ferritin levels, hemoglobin levels, and transfusion requirements. By addressing this gap, the study aims to provide novel insights into persistent thrombocytosis and its implications for patient management, complications, and the need for antiplatelet prophylaxis, ultimately improving care both locally and globally.

## 2. Methods

### 2.1. Study design and setting

This study was a retrospective cohort study conducted at Al-Bashir Hospital, a referral center specializing in β-thalassemia care in Amman, Jordan. The study period spanned from November 2018 to May 2024. It was designed and reported in accordance with the Strengthening the Reporting of Observational Studies in Epidemiology criteria,^[14]^ ensuring rigor, clarity, and completeness in reporting the outcomes of surgical interventions among β-thalassemia patients.

### 2.2. Study population

The study population consisted of 22 patients diagnosed with β-thalassemia major who underwent splenectomy. The sample size was considered acceptable for descriptive and comparative analyses, given the rarity of this patient population. The dataset was obtained from the electronic medical records of Al-Bashir Hospital. Inclusion criteria required patients to have a confirmed diagnosis of β-thalassemia major and complete pre- and post-splenectomy medical records. Patients diagnosed with other forms of thalassemia, such as β-thalassemia intermedia or β-thalassemia minor, or those who underwent splenectomy for reasons unrelated to β-thalassemia major, were excluded. In addition, patients with conditions that could potentially confound platelet or transfusion outcomes, such as autoimmune disorders or malignancies, were excluded.

### 2.3. Data collection

Data were collected from the hospital’s electronic medical records using a standardized chart review form. The dataset comprised demographic variables such as age, gender, and year of surgery, alongside clinical data, including indications for splenectomy, spleen size, pre- and post-splenectomy blood transfusion requirements, hemoglobin levels, platelet counts, and comorbidities. Laboratory data included ferritin levels, liver and kidney function tests, and vitamin B12 and vitamin D levels. Information on post-splenectomy treatments, such as prescriptions for aspirin, hydroxyurea, and penicillin, as well as the administration of pneumococcal vaccination, was also collected. Data were handled with strict confidentiality, using industry-standard encryption methods during storage and transmission, and were accessed only by authorized personnel to ensure privacy and data security.

### 2.4. Outcome measures

The primary outcome of the study was the persistence of thrombocytosis, measured by platelet counts (cells/mm^3^) at various intervals before and after splenectomy. Secondary outcomes included changes in blood transfusion requirements, assessed as the volume of blood transfused (mL/kg/yr) and the interval between transfusions, as well as changes in hemoglobin levels (g/dL).

### 2.5. Statistical analysis

Data analysis was performed using IBM SPSS Statistics version 26. Continuous variables were summarized as means and standard deviations, while categorical variables were expressed as frequencies and percentages. Pre- and post-splenectomy outcomes, including platelet counts, hemoglobin levels, and transfusion requirements, were compared using paired-samples *t* tests, as the data were normally distributed according to the Shapiro–Wilk test (*P* > .05). A *P* value of <.05 was considered statistically significant. All variables collected for each study participant were available, and no missing data were encountered.

### 2.6. Ethical considerations

Ethical approval for this study was granted by the Institutional Review Board of the Jordanian Ministry of Health (Approval No. 7365/2024). The research was conducted in compliance with the ethical guidelines set forth by the committee and adhered to the principles outlined in the Declaration of Helsinki (1964) and its subsequent revisions. As this was a retrospective study utilizing de-identified electronic medical records, obtaining individual consent from participants was deemed unnecessary. All data were managed with strict confidentiality, employing industry-standard encryption for secure storage and transmission, and were used exclusively for research purposes.

## 3. Results

### 3.1. General characteristics of the study population

The study included 22 patients diagnosed with β-thalassemia major, identified from Al-Bashir Hospital’s database, all of whom underwent splenectomy at the hospital or in private facilities. All patients were Jordanian nationals diagnosed with β-thalassemia major. Table 1 illustrates the demographic characteristics of the patients. The majority were female (n = 14, 63.64%), while males accounted for a smaller proportion (n = 8, 36.36%). The mean age ± standard deviation of the patients was 24.71 ± 8.44 years, with birth years ranging from 1977 to 2010. Most patients underwent splenectomy at Al-Bashir Hospital (n = 19, 86.36%), while 3 patients (13.64%) underwent the procedure at private hospitals.

**Table 1:** General characteristics of patients (N = 22).

	N	%
Gender		
Male	8	36.36
Female	14	63.64
Nationality		
Jordanian	22	100
Place of splenectomy		
Al-Bashir Hospital	19	86.36
Private hospital	3	13.64
Type of thalassemia		
Beta-thalassemia major	22	100
Blood group		
A+	6	27.27
AB−	1	4.55
B+	4	18.18
O−	3	13.64
O+	8	36.36
	M	±SD
Age at surgery (yr)	24.71	8.44
Yr of birth	1996.77	9.2
Yr of splenectomy	2020.91	1.44
Weight at surgery (kg)	53.24	6.98
Spleen size at surgery (cm)	19.34	2.15

% = percentage, M = mean, N = number of patients, SD = standard deviation.

#### 3.1.1. Indications for splenectomy

Indications for splenectomy included annual blood transfusion requirements exceeding 250 mL/kg (n = 20), hypersplenism (n = 17), and massive splenomegaly (n = 5). The average weight of the patients at the time of surgery was 53.24 ± 6.98 kg. The spleen size at surgery ranged from 14.8 to 20.6 cm, with an average size of 19.34 ± 2.15 cm. The blood groups of the patients are shown in Table 1.

### 3.2. Post-splenectomy medical treatments

Post-splenectomy, medical treatment for patients included 4 components, as outlined in Table 2. Penicillin was prescribed for all patients (n = 22, 100%), while half of the patients (n = 11, 50%) were prescribed aspirin, and 36.36% (n = 8) received hydroxyurea. In addition, the pneumococcal vaccination was administered to all patients (n = 22, 100%).

**Table 2:** Drugs and blood transfusions among patients (N = 22).

Drugs	N	%			
Aspirin	11	50			
Hydroxyurea	8	36.36			
Penicillin	22	100			
Pneumococcal vaccine	22	100			
	Before splenectomy		After splenectomy		*P* value
	M	±SD	M	±SD	
Blood transfusion requirements (mL/kg/yr)	295.95	68.24	170.21	54.65	**<.001**
Blood transfusion interval (d)	17.14	4.49	27.45	10.5	**<.001**

*P* values that were statistically significant are shown in bold.

% = percentage, M = mean, N = number of patients, SD = standard deviation.

The average blood transfusion requirement decreased significantly by 42.49%, from 295.95 ± 68.24 mL/kg/yr pre-splenectomy to 170.21 ± 54.65 mL/kg/yr post-splenectomy (*P* < .001). The interval between blood transfusions increased by 60.15%, from 17.14 ± 4.49 days pre-splenectomy to 27.45 ± 10.50 days post-splenectomy (*P* < .001).

### 3.3. Platelet counts

Average platelet counts were measured over 15 intervals during the year preceding splenectomy, as shown in Table S1. The average counts ranged from 249.64 ± 216.00 to 371.91 ± 428.99 × 10^9^/L. Upon admission, 5 readings were taken, with mean counts ranging from 262.45 ± 142.14 to 535.32 ± 191.78 × 10^9^/L. Similarly, 15 readings were taken post-splenectomy, revealing persistent thrombocytosis.

The first post-splenectomy reading showed a mean of 965.36 ± 413.50 × 10^9^/L, which gradually decreased over time, reaching 843.23 ± 320.08 × 10^9^/L by the 15th interval, 1 year post-splenectomy. Across the 15 measurement intervals, mean platelet counts increased from 355.55 ± 303.07 × 10^9^/L pre-splenectomy to 965.36 ± 413.50 × 10^9^/L immediately post-splenectomy, and then stabilized at 843.23 ± 320.08 × 10^9^/L after 1 year, as detailed in Table S1 and Figure S1. This sustained elevation confirms persistent thrombocytosis. Importantly, no thrombotic or hemorrhagic events were observed during follow-up.

### 3.4. Hemoglobin levels

Average hemoglobin levels (g/dL) were assessed over 15 intervals during the year pre-splenectomy, 5 intervals upon admission for splenectomy, and 15 intervals in the year post-splenectomy, as shown in Table 3 and Figure S2. Supplemental Digital Content.

**Table 3:** Average hemoglobin counts (g/dL) 1 year pre- and post-splenectomy and on admission (N = 22).

Intervals	Pre-splenectomy		On admission		Post-splenectomy	
	M	±SD	M	±SD	M	±SD
1	8.47	1.43	10.70	1.92	9.65	1.49
2	8.42	1.20	10.70	2.21	9.28	1.18
3	7.75	1.20	10.25	1.69	9.10	1.25
4	7.84	1.48	9.90	1.75	9.10	1.00
5	8.13	1.09	9.43	0.99	8.91	0.96
6	8.05	1.42	–	–	9.00	0.95
7	8.48	1.17	–	–	9.02	0.89
8	8.17	1.31	–	–	9.14	0.67
9	8.17	1.49	–	–	9.07	0.90
10	8.19	1.45	–	–	9.36	1.00
11	8.27	1.54	–	–	9.05	0.92
12	8.51	1.41	–	–	9.31	0.79
13	8.48	1.55	–	–	9.37	0.91
14	8.58	1.20	–	–	9.02	1.06
15	8.43	1.34	–	–	9.22	0.89

M = mean, SD = standard deviation.

Pre-splenectomy, mean hemoglobin levels remained relatively stable, ranging from 7.75 ± 1.20 to 8.58 ± 1.20 g/dL. A noticeable increase in mean hemoglobin levels was observed upon admission, reaching 10.70 ± 1.92 g/dL. Post-splenectomy, mean hemoglobin levels ranged from 8.91 ± 0.96 to 9.65 ± 1.49 g/dL, reflecting a consistent improvement compared with pre-splenectomy levels.

### 3.5. Ferritin levels and laboratory trends

#### 3.5.1. Ferritin levels

**Figure 1.**
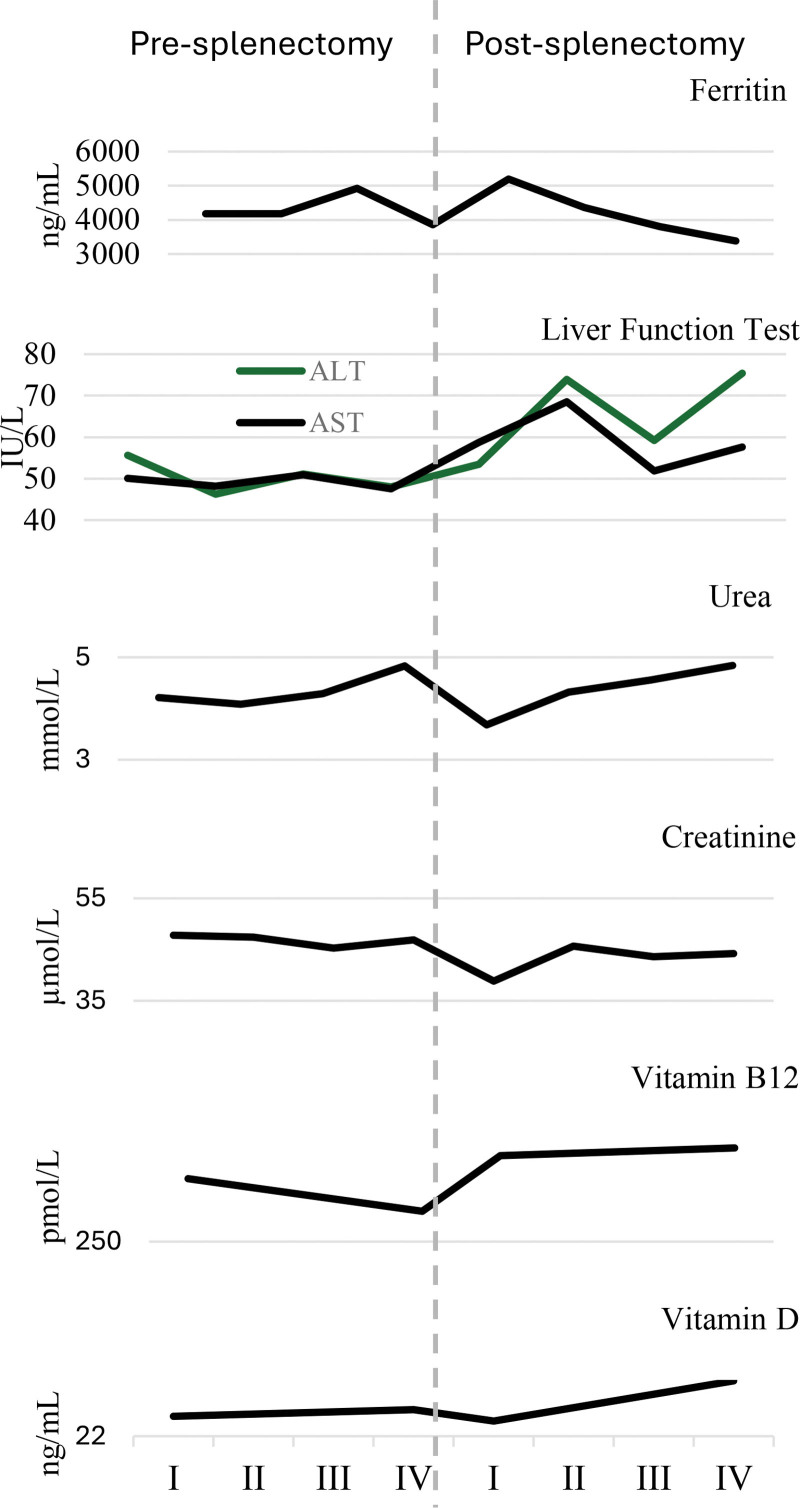
Average laboratory results 1 year pre- and post-splenectomy (N = 22). ALT = alanine aminotransferase, AST = aspartate aminotransferase.

Ferritin levels (ng/mL) were evaluated over 4 intervals during the year pre-splenectomy and 4 intervals during the year post-splenectomy. Trends in mean laboratory results are shown in Table S2 and Figure 1. Mean ferritin levels peaked during the third pre-splenectomy interval at 4918.86 ± 3479.38 ng/mL and gradually decreased post-splenectomy, reaching a minimum mean of 3381.73 ± 2321.91 ng/mL by the final interval.

#### 3.5.2. Liver function tests

Liver function tests (IU/L), including alanine aminotransferase (ALT) and aspartate aminotransferase, remained relatively stable pre-splenectomy. Post-splenectomy, mean ALT levels fluctuated between 53.47 ± 51.05 IU/L and 75.40 ± 68.56 IU/L, while aspartate aminotransferase levels ranged from 51.81 ± 30.48 IU/L to 68.52 ± 106.76 IU/L.

#### 3.5.3. Kidney function tests

Urea (mmol/L) and creatinine (µmol/L) levels were assessed to evaluate kidney function. Mean urea levels remained consistent pre- and post-splenectomy, measuring 4.83 ± 1.80 mmol/L and 4.84 ± 1.72 mmol/L, respectively. Mean creatinine levels were relatively stable, except for a transient drop from 46.89 ± 22.27 µmol/L in the final pre-splenectomy reading to 38.84 ± 15.91 µmol/L immediately post-splenectomy. Subsequently, mean creatinine levels returned to their pre-splenectomy baseline, reaching 44.23 ± 17.18 µmol/L in the final post-splenectomy reading.

#### 3.5.4. Vitamins B12 and D

Vitamin B12 (pmol/L) and vitamin D (ng/mL) levels were measured twice pre-splenectomy and twice post-splenectomy. Mean vitamin B12 levels increased from 294.45 ± 116.76 pmol/L pre-splenectomy to 375.68 ± 268.34 pmol/L immediately post-surgery. Vitamin D levels showed a slight increase, with mean values rising from 23.41 ± 9.53 ng/mL pre-splenectomy to 25.94 ± 10.99 ng/mL 1 year post-splenectomy.

## 4. Discussion

This retrospective cohort study evaluates the long-term outcomes of β-thalassemia patients post-splenectomy and is the first of its kind in Jordan. The cohort included 22 β-thalassemia patients, with a mean age of 24.71 ± 8.44 years, who underwent splenectomy between 2018 and 2024. The findings provide valuable insights into persistent thrombocytosis, hemoglobin levels, transfusion dependency, and iron overload management, highlighting significant clinical implications and challenges in patient care.

Persistent thrombocytosis was observed in all patients, with mean platelet counts peaking at 965.36 ± 413.50 × 10^9^/L immediately post-surgery and declining slightly to 843.23 ± 320.08 × 10^9^/L at the final follow-up. These findings align with results reported in the literature, where platelet counts significantly increased after splenectomy at 1 and 5 years compared with baseline values.^[15]^ When compared with non-splenectomized patients, post-splenectomized thalassemia intermedia patients exhibited the highest platelet counts.^[16]^ For instance, a study conducted in Egypt (2014) reported platelet counts averaging 644.7 ± 299.4 × 10^9^/L over a 6.26-year follow-up period, with 1 case of portal vein thrombosis occurring 12 months post-surgery.^[17]^ Similarly, a study in India observed an average post-splenectomy platelet count of 591.2 ± 451.0 × 10^9^/L, with 2 patients developing thrombotic complications requiring long-term anticoagulation therapy.^[18]^ Thrombocytosis was present in 87% of splenectomized patients, with 7 (17.9%) exhibiting platelet counts exceeding 1000 × 10^9^/L, and 2 (5.1%) experiencing thrombosis.^[19]^ Another study highlighted a significant rise in platelet counts post-splenectomy, with 5 (4.8%) patients developing thrombosis in the absence of aspirin prophylaxis.^[20]^ In contrast, no patients in our study experienced thrombotic or hemorrhagic events during follow-up, suggesting that routine prophylactic measures and close postoperative monitoring may effectively mitigate such risks.

The spleen’s role explains these observations. It contains red pulp, which filters the blood, and white pulp, which is responsible for immunity against encapsulated pathogens.^[21]^ Its central role in platelet destruction and sequestration accounts for thrombocytosis in asplenic or hyposplenic patients.^[22,23]^ Platelet counts reportedly increase by 30% to 100% post-splenectomy, peaking within 7 to 20 days and stabilizing over weeks, months, or even years in some cases.^[24]^ Consequently, postoperative thrombocytosis is observed in up to 90% of splenectomized patients,^[25,26]^ with a higher risk of thrombotic events due to the resulting hypercoagulable state, characterized by increased platelet levels, elevated thrombin generation, and reduced anticoagulant activity.^[27]^

Management of thrombocytosis is critical in thalassemia patients post-splenectomy, in accordance with the Thalassaemia International Federation guidelines.^[28]^ All patients in this study received prophylactic antibiotics and vaccinations against encapsulated pathogens. Aspirin, an antiplatelet agent, was prescribed to all patients to mitigate thrombotic risk; however, adherence was suboptimal, with only 11 patients complying. Similarly, hydroxyurea, another medication used to manage elevated platelet counts,^[29]^ was prescribed for patients with platelet counts exceeding 1000 × 10^9^/L, but only 8 adhered to the regimen, and 5 refused treatment.

Suboptimal adherence to aspirin and hydroxyurea may compromise prophylactic efficacy and increase thrombotic risk. Addressing this challenge requires enhanced patient education, counseling, and structured follow-up protocols to ensure consistent compliance and improved long-term outcomes. Studies on medication nonadherence in the region have shown rates exceeding 25%, attributed to several factors such as forgetfulness, low educational level, fear of adverse drug effects, absence from home at medication time, and frequent changes in treatment regimens.^[30–32]^ Addressing these barriers through patient education programs and community health initiatives is essential to optimizing adherence and improving clinical outcomes.

Hemoglobin levels significantly improved post-splenectomy due to reduced red blood cell destruction by the spleen. Pre-splenectomy, hemoglobin levels remained stable, followed by a noticeable increase during admission and a sustained rise post-splenectomy, reaching a mean of 9.28 g/dL, ranging from 8.91 ± 0.96 g/dL at the beginning to 9.65 ± 1.49 g/dL at the final follow-up. These findings are consistent with a 2018 study from Pakistan, which reported an average post-splenectomy hemoglobin level of 9.2 g/dL.^[33]^ Other studies have similarly demonstrated significant increases in pre-transfusion hemoglobin levels 1 and 5 years post-splenectomy.^[15]^ This significant improvement in hemoglobin levels underscores the efficacy of splenectomy in enhancing oxygen-carrying capacity and alleviating the clinical burden of anemia.

Patients with severe thalassemia often require regular blood transfusions every few weeks to maintain hemoglobin levels between 9 and 10 g/dL, ensuring overall well-being.^[34]^ Splenectomy aims to reduce transfusion dependency, which was achieved in this study.

Blood transfusion requirements decreased by 42.49%, from 295.95 ± 68.24 mL/kg/yr pre-splenectomy to 170.21 ± 54.65 mL/kg/yr post-splenectomy. This statistically significant reduction persisted throughout follow-up and was accompanied by a marked increase in transfusion interval, from 17.14 ± 4.49 days to 27.45 ± 10.5 days. These changes substantially reduce the burden on both patients and healthcare systems while improving patients’ overall quality of life. Our findings are consistent with the literature, which supports the long-term benefits of splenectomy in reducing transfusion requirements, with some patients even becoming transfusion-independent.^[15,18,35,36]^ Furthermore, previous studies have emphasized the stability of transfusion needs following the initial postoperative decrease during the first year.^[37]^

Another notable outcome is the improvement in iron overload, primarily attributed to reduced transfusion dependency and overall iron intake.^[16,37]^ Ferritin levels, initially elevated (mean 4180.64 ng/mL), gradually decreased to 3857.95 ng/mL by the final follow-up. This trend aligns with previous reports in the literature, where ferritin and ALT levels significantly improved following splenectomy.^[35]^ Addressing iron overload remains crucial for enhancing patient quality of life and minimizing long-term complications, including hepatic and endocrine organ damage.

This study highlights the critical role of splenectomy in improving hematological parameters and overall quality of life for patients with β-thalassemia major. Persistent thrombocytosis remains a concern, emphasizing the need for regular monitoring and effective management strategies to prevent thrombotic complications. The absence of thrombotic events in our cohort reinforces the potential safety of splenectomy when appropriate prophylaxis and monitoring protocols are implemented. Furthermore, targeted efforts to improve adherence to aspirin and hydroxyurea should be prioritized to sustain favorable outcomes and reduce preventable risks. Addressing medication adherence through structured and targeted interventions is essential to optimizing long-term outcomes.

Despite its significant findings, this study has several limitations. Its retrospective design inherently introduces risks of selection and documentation bias, which may affect data accuracy. In addition, the small sample size limits statistical power and generalizability, particularly when assessing trends such as thrombocytosis or transfusion reduction. Potential confounding factors, including variations in baseline characteristics, differences in adherence to prescribed medications, and individual physiological responses to splenectomy, may also have influenced the outcomes. Furthermore, the absence of a control group restricts the ability to establish causality between splenectomy and the observed hematologic and clinical improvements.

To address these limitations, future research should focus on large-scale, multicenter prospective studies with well-defined control groups. Such studies could more accurately evaluate the long-term effects of splenectomy on β-thalassemia while accounting for key variables such as adherence to postoperative management protocols, chelation therapy, and regional differences in care practices. In addition, implementing standardized data collection methods across similar clinical settings could help minimize bias and enhance the reliability and reproducibility of future findings.

## 5. Conclusion

This study demonstrates the long-term benefits of splenectomy in patients with β-thalassemia major, including improved hemoglobin levels, reduced transfusion requirements, and decreased iron overload. The presence of persistent thrombocytosis in all patients underscores the importance of vigilant monitoring and effective thrombosis prevention strategies. Importantly, no thrombotic events were observed during the 1-year follow-up, supporting the safety of splenectomy when appropriate prophylaxis and regular follow-up are maintained. Enhancing adherence to medications, particularly aspirin and hydroxyurea, remains crucial to minimize complications and sustain favorable outcomes. Long-term follow-up and multicenter prospective studies are recommended to validate these findings and further assess the durability and safety of hematologic and clinical outcomes following splenectomy.

## Author contributions

**Conceptualization:** Aisheh Alesufi, Saif Aburumman.

**Methodology:** Aisheh Alesufi, Naser Aldain A. Abu Lehyah.

**Data curation:** Naser Aldain A. Abu Lehyah, Nour Fakih, Lama Al-Karmi.

**Project administration:** Dima Abu Nasrieh.

**Formal analysis:** Saif Aburumman.

**Visualization:** Saif Aburumman.

**Supervision:** Qasem Shersheer.

**Writing – original draft:** Naser Aldain A. Abu Lehyah, Dima Abu Nasrieh, Haneen A. Banihani, Zahraa Zibara, Nour Fakih, Qasem Shersheer.

**Writing – review & editing:** Haneen A. Banihani, Zahraa Zibara, Nour Fakih, Maram A. Al Nawaiseh.








