# Supplementary material for: Persistent thrombocytosis in β-thalassemia post-splenectomy: A STROBE-compliant retrospective cohort study at a Jordanian referral center
Source: Medicine (Baltimore). 2026 May 15;105(20):e48717. doi: 10.1097/MD.0000000000048717 (PMC13183013; doi:10.1097/MD.0000000000048717)
Supplement: Supplementary file 1 [file medi-105-e48717-s001.docx]

**Table S1.** Average Platelet Counts (×10⁹/L) One Year Pre- and Post-Splenectomy and on Admission (N = 22)
Note: Platelet counts are expressed in ×10⁹/L (1 × 10⁹/L = 1,000,000 cells/mm³).
M: Mean; SD: Standard Deviation.

| Intervals | Pre-splenectomy | | On admission | | Post-splenectomy | |
| --- | --- | --- | --- | --- | --- | --- |
|  | **M** | **± SD** | **M** | **± SD** | **M** | **± SD** |
| 1 | 355.55 | 303.07 | 262.45 | 142.14 | 965.36 | 413.50 |
| 2 | 332.00 | 318.86 | 405.14 | 271.23 | 940.73 | 307.52 |
| 3 | 331.59 | 315.41 | 479.86 | 284.28 | 943.82 | 298.39 |
| 4 | 371.91 | 428.99 | 460.64 | 203.64 | 907.91 | 248.23 |
| 5 | 336.23 | 330.63 | 535.32 | 191.78 | 858.64 | 253.05 |
| 6 | 319.73 | 242.89 |  |  | 873.36 | 312.54 |
| 7 | 317.64 | 237.79 |  |  | 813.36 | 273.91 |
| 8 | 251.18 | 200.28 |  |  | 809.55 | 267.03 |
| 9 | 316.23 | 249.71 |  |  | 809.68 | 323.35 |
| 10 | 288.36 | 261.09 |  |  | 871.86 | 370.02 |
| 11 | 279.23 | 235.14 |  |  | 764.68 | 314.51 |
| 12 | 277.00 | 275.25 |  |  | 829.68 | 343.79 |
| 13 | 249.64 | 216.00 |  |  | 826.82 | 303.65 |
| 14 | 275.09 | 230.21 |  |  | 892.18 | 320.40 |
| 15 | 260.36 | 230.23 |  |  | 843.23 | 320.08 |
