# Supplementary material for: Persistent thrombocytosis in β-thalassemia post-splenectomy: A STROBE-compliant retrospective cohort study at a Jordanian referral center
Source: Medicine (Baltimore). 2026 May 15;105(20):e48717. doi: 10.1097/MD.0000000000048717 (PMC13183013; doi:10.1097/MD.0000000000048717)
Supplement: Supplementary file 2 [file medi-105-e48717-s002.docx]

**Figure S1.** Average Platelet Counts (cells/mm³) One Year Pre- and Post-Splenectomy and on Admission (N = 22)
