# Supplementary material for: Persistent thrombocytosis in β-thalassemia post-splenectomy: A STROBE-compliant retrospective cohort study at a Jordanian referral center
Source: Medicine (Baltimore). 2026 May 15;105(20):e48717. doi: 10.1097/MD.0000000000048717 (PMC13183013; doi:10.1097/MD.0000000000048717)
Supplement: Supplementary file 4 [file medi-105-e48717-s004.docx]

**Table S2.** Laboratory Results Means One Year Pre- and Post-Splenectomy and on Admission (N = 22)
Note: All laboratory values are expressed in standard SI units.

M: Mean; SD: Standard Deviation.

|  |  | Ferritin | | ALT | | AST | | Urea | | Creatinine | | Vit B12 | | Vit D | |
| --- | --- | --- | --- | --- | --- | --- | --- | --- | --- | --- | --- | --- | --- | --- | --- |
|  |  | ng/mL | | IU/L | | IU/L | | mmol/L | | µmol/L | | pmol/L | | ng/mL | |
|  | Intervals | **M** | **± SD** | **M** | **± SD** | **M** | **± SD** | **M** | **±SD** | **M** | **± SD** | **M** | **± SD** | **M** | **± SD** |
| Pre-splenectomy | I | 4180.64 | 2051.32 | 55.62 | 47.75 | 50.05 | 32.60 | 4.21 | 1.24 | 47.80 | 17.22 | 342.18 | 164.89 | 23.41 | 9.53 |
|  | II | 4174.50 | 2951.08 | 46.25 | 49.60 | 48.14 | 37.22 | 4.08 | 1.58 | 47.38 | 17.57 |  |  |  |  |
|  | III | 4918.86 | 3479.38 | 51.14 | 40.68 | 50.92 | 30.62 | 4.29 | 1.49 | 45.31 | 19.53 |  |  |  |  |
|  | IV | 3857.95 | 2368.25 | 47.97 | 62.81 | 47.54 | 39.08 | 4.83 | 1.80 | 46.89 | 22.27 | 294.45 | 116.76 | 23.90 | 10.25 |
| Post-splenectomy | I | 5194.23 | 3171.19 | 53.47 | 51.05 | 58.73 | 86.60 | 3.68 | 1.27 | 38.84 | 13.54 | 375.68 | 268.34 | 23.08 | 12.31 |
|  | II | 4357.14 | 3288.93 | 73.92 | 96.48 | 68.52 | 106.76 | 4.32 | 1.44 | 45.68 | 15.91 |  |  |  |  |
|  | III | 3804.77 | 2334.94 | 59.21 | 40.04 | 51.81 | 30.48 | 4.56 | 1.13 | 43.61 | 13.78 |  |  |  |  |
|  | IV | 3381.73 | 2321.91 | 75.40 | 68.56 | 57.59 | 43.44 | 4.84 | 1.72 | 44.23 | 17.18 | 387.18 | 154.32 | 25.94 | 10.99 |
